# Decreased expression of MUC2 due to a decrease in the expression of lectins and apoptotic defects in colitis patients

**DOI:** 10.1016/j.bbrep.2019.100655

**Published:** 2019-06-05

**Authors:** Agung A. Wibowo, Bambang Pardjianto, Sutiman B. Sumitro, Nia Kania, Kusworini Handono

**Affiliations:** a Doctoral Program in Medicine, Faculty of Medicine, Universitas Brawijaya, Malang, East Java, Indonesia; b Department of Surgery, Ulin General Hospital, Medical Faculty, Lambung Mangkurat University, Banjarmasin, South Kalimantan, Indonesia; c Department of Plastic Surgery, Faculty of Medicine Universitas Brawijaya/dr. Saiful Anwar General Hospital, Malang, East Java, Indonesia; d Department of Biology, Faculty of Sciences, Universitas Brawijaya Malang, East Java, Indonesia; e Department of Pathology, Ulin General Hospital, Medical Faculty, Lambung Mangkurat University, Banjarmasin, South Kalimantan, Indonesia; f Department of Clinical Pathology, Faculty of Medicine Universitas Brawijaya/dr.Saiful Anwar General Hospital, Malang, East Java, Indonesia

**Keywords:** Mucin, Inflammation, Apoptosis, Lectin, Colon

## Abstract

**Introduction:**

The involvement of mucin, lectin, and apoptosis in colitis is still unclear. This study aimed to investigate changes in MUC2 expression, inflammation, and changes in lectin expression in colitis patients.

**Methods:**

A total of 17 patients were divided into two groups including 11 hemorrhoid patients as a control group and 6 colitis patients. MUC2 mutation analysis was carried out using immunofluorescent and FISH techniques. Assessment of caspase-3, Ki-67, NF-kB, and lectin expressions was also carried out by immunofluorescent technique then analyzed by confocal laser scanning microscope.

**Results:**

The MUC2, caspase-3, and lectin expressions were significantly lower in the colitis group than in the control group (p < 0.05).

**Conclusions:**

It was concluded that in colitis there was a change in MUC2 expression due to changes in lectins accompanied by apoptotic defects.conclusion

## Highlights

• Clinical molecular study on colitis patients.
• Caspase-3 expression decreased significantly in colitis patients.
• MUC-2 expression decreased significantly in the colitis.
• The lectin expression in colitis was significantly lower.

## 1 Introduction

Colitis is a chronic inflammatory disease, characterized by the presence of plasmacytosis in the lamina propria colon, due to unknown etiology [1,2]. The manifestations of this disease are heterogeneous due to complex immunopathological processes, including immune response dysregulation, aberrant cytokine secretion, changes in barrier function, and intestinal microbiota [3,4]. In western countries, the prevalence of colitis is 200–250 per 100,000 inhabitants [5]. In Africa and the Middle East, the tendency for colitis follow the pattern in developed countries. This phenomenon is also found in Asia [7].

MUC2 is a mucin layer in the gastrointestinal tract, along with epithelial cells that will form a barrier to toxic substances. MUC2 is secreted high in the colon epithelium cells of humans, mice and mice. MUC2 is stored in the apical granule of goblet cells as a morphological determinant of goblet cells. In MUC2-deficient mice, colon inflammation will occur and contribute to the development of colitis induction [8]. Nonetheless, there is still controversy in the expression of MUC2 in colitis patients, in the form of decreased expression [9], as well as a moderate increase in MUC2 expression compared to controls [10]. Even for mRNA levels, there was no difference between colitis and controls [11].

Lectin is a glycoprotein that is able to recognize and bind irreversibly with carbohydrates from the glycoconjugate complex [12]. Lectin is a glycan binding protein [13]. The main function of glycan is the protection of core proteins from degradation by proteases [14]. In colitis, glycan changes are found which result in damage to the mucous barrier and the appearance of inflammasome [15,16]. Acetylation of glycoproteins is a mucosal recovery marker for colitis patients [17]. Until now, as far as we know, there have not been many studies evaluating the changes in lectins in colitis.

The contribution of inflammation in the development of colitis is still controversial. About 40% of patients do not respond to anti-TNF-α treatment [18,19]. Apoptosis can be divided into two pathways, namely the mitochondrial intrinsic pathway and extrinsic death receptor. Both of these pathways are interrelated and can influence each other. Both of these pathways also activate caspase, a protease that targets cellular proteins, triggering cell disassembly [20]. A previous study has revealed a decrease in intrinsic apoptosis in colitis compared to controls [21]. The role of apoptosis in colitis is also still controversial. Therefore, this study will investigate changes in MUC2 expression, inflammation, and changes in lectin expression in colitis patients.

## 2 Material and methods

### 2.1 Tissue

There are two groups, including the control group (hemorrhoid patient) (11 patients) and the group of colitis patients (6 patients). Criteria for patients are determined through clinical, endoscopic, and histological examinations. Tissue is obtained from the biopsy. For controls, there was no prior history of inflammatory bowel disease, endoscopic and histologic analysis showed normal colon. Determination of healthy tissue from biopsy of hemorrhoid patients is carried out by a pathologist. For colitis patients, endoscopic, and histological analysis shows moderate to severe inflammation. A biopsy is performed in the sigmoid region (30 cm from the anal ring) [11]. The use of human tissue has received ethical approval from the Research Ethics Committee, Faculty of Medicine, Banjarmasin, South Kalimantan, Indonesia.

### 2.2 Labelling immunofluorescence

Labelling immunofluorescence was carried out according to the protocol in the previous study [22]. Antibodies used include anti-Caspase-3 mouse monoclonal antibody (Santa Cruz Biotechnology, Dallas, Texas, US), anti Ki67 rabbit polyclonal antibody (Santa Cruz Biotechnology, Dallas, Texas, US), anti-Mucin 2 rabbit polyclonal antibody (Santa Cruz Biotechnology, Dallas, Texas, US), anti-NFkB mouse monoclonal antibody (Santa Cruz Biotechnology, Dallas, Texas, US). Secondary antibodies include goat anti-Rabbit IgG-FITC (Santa Crus Biotechnology, Dallas, Texas, US) (1:1000), and goat anti-Mouse IgG-Rhod (Santa Cruz Biotechnology, Dallas, Texas, US).

### 2.3 FISH of MUC2 (5′-TCC AAT GGG AAC ATC AGGATA CAT GGT GGC-3′)

First, prepared 40 ml of 0.01 N HCL (0.85% NaCl) in the staining jar on the waterbath temperature 37 °C. The slides were incubated at 90 °C for 25 min. Slides are depreciated with xylene for 15 min, repeated twice. Then dehydrated with absolute ethanol for 5 min, repeated 2 times. The slides are air dried. Prepared 10 mM citric acid incubated in waterbath temperature 80 °C. The slides were incubated in citric acid for 55 min. Prepared 80 mg of Pepsin dissolved with 0.01 N HCl (0.2% Pepsin). Slide incubated in pepsin solution for 30 min. Slide is washed with 70% ethanol for 30 s. The slides are air dried and checked for differences before and after incubation of the Pepsin solution. Slides are dehydrated with 70% ethanol, 85%, and 100% for 2 min each. Then the slides were dried, then continued with the hybridization step. Prepared 10  μl mix per slide probe. The hybridization was carried out by the following process: 83 °C denaturation for 3 min, 37 °C hybridization for 24–48 h, then washed with 0.3% Igepal, CA-630, Sigma (or NP40)/0.4XSSC @ 73 °C for 2 min. Finally, it was washed with 0.1% IgEpal CA-630, Sigma (or NP40)/2XSSC at room temperature for 90 s.

### 2.4 Ethics

In this study, the ethics committee approval was given by the Health Research Ethics Committee of the Faculty of Medicine, Banjarmasin, South Kalimantan, Indonesia. (No.30/KEPK-FK UNLAM/01/2019).

### 2.5 Statistical analysis

Data are presented as mean ± SD and differences between treatment groups are analyzed by the unpaired *t*-test. The analysis was carried out with the SPSS 23.0 statistical package for Windows program. The probability value (*p* < 0.05) is stated to be significantly different.

## 3 Results

In this study, eleven healthy colon tissues were isolated from hemmorhoid patients. For colitis patients, six colon tissue samples were obtained.

**Fig. 1:**
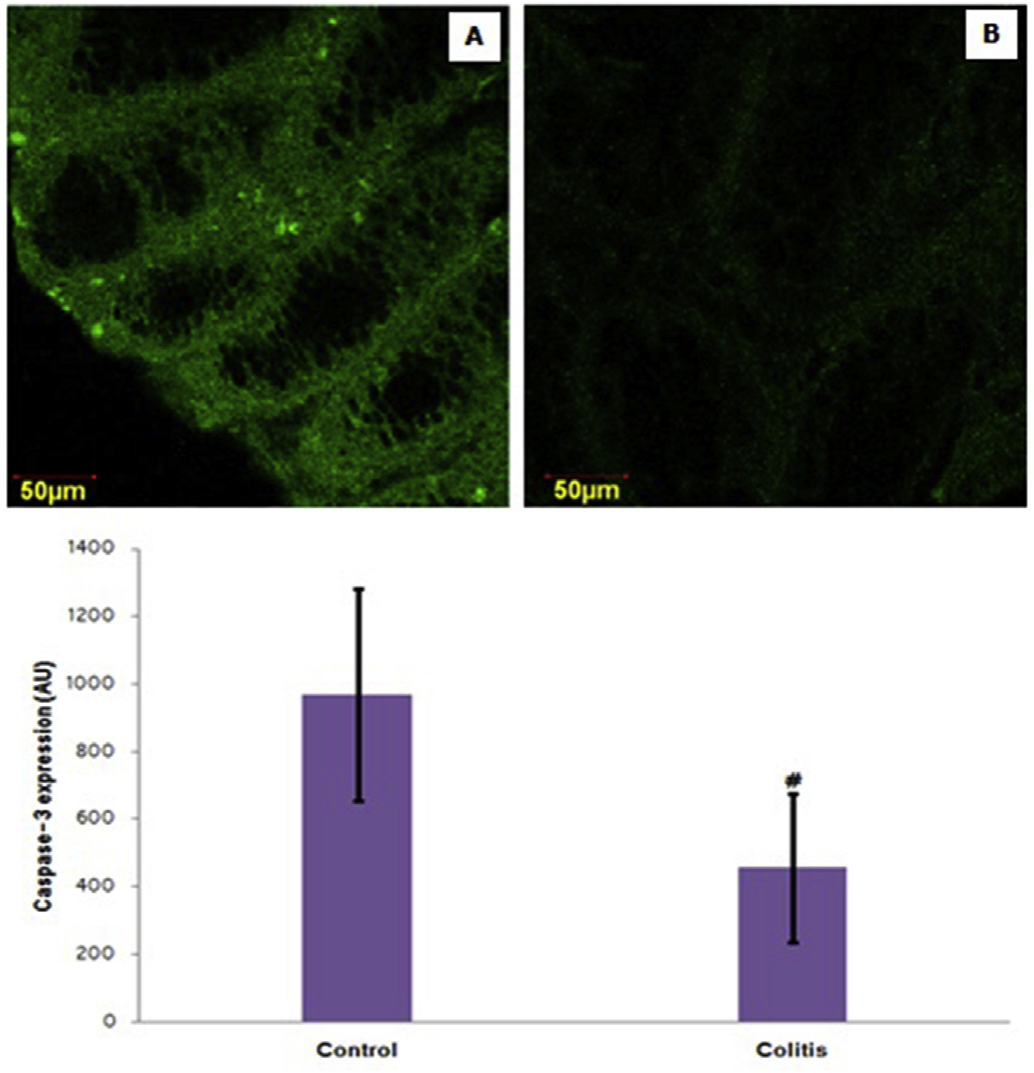
Representative micrograph of caspase-3 expression in healthy colon tissue (A) and colitis patients (B). (FITC staining at 400x magnification by a confocal laser scanning microscope) (Top figure). The expression of caspase-3 in control and colitis. Note: Value are presented as mean ± standard of deviation; AU: arbitrary units; ^#^: *p* < 0.05 in comparison with the control group (Bottom figure).

**Fig. 2:**
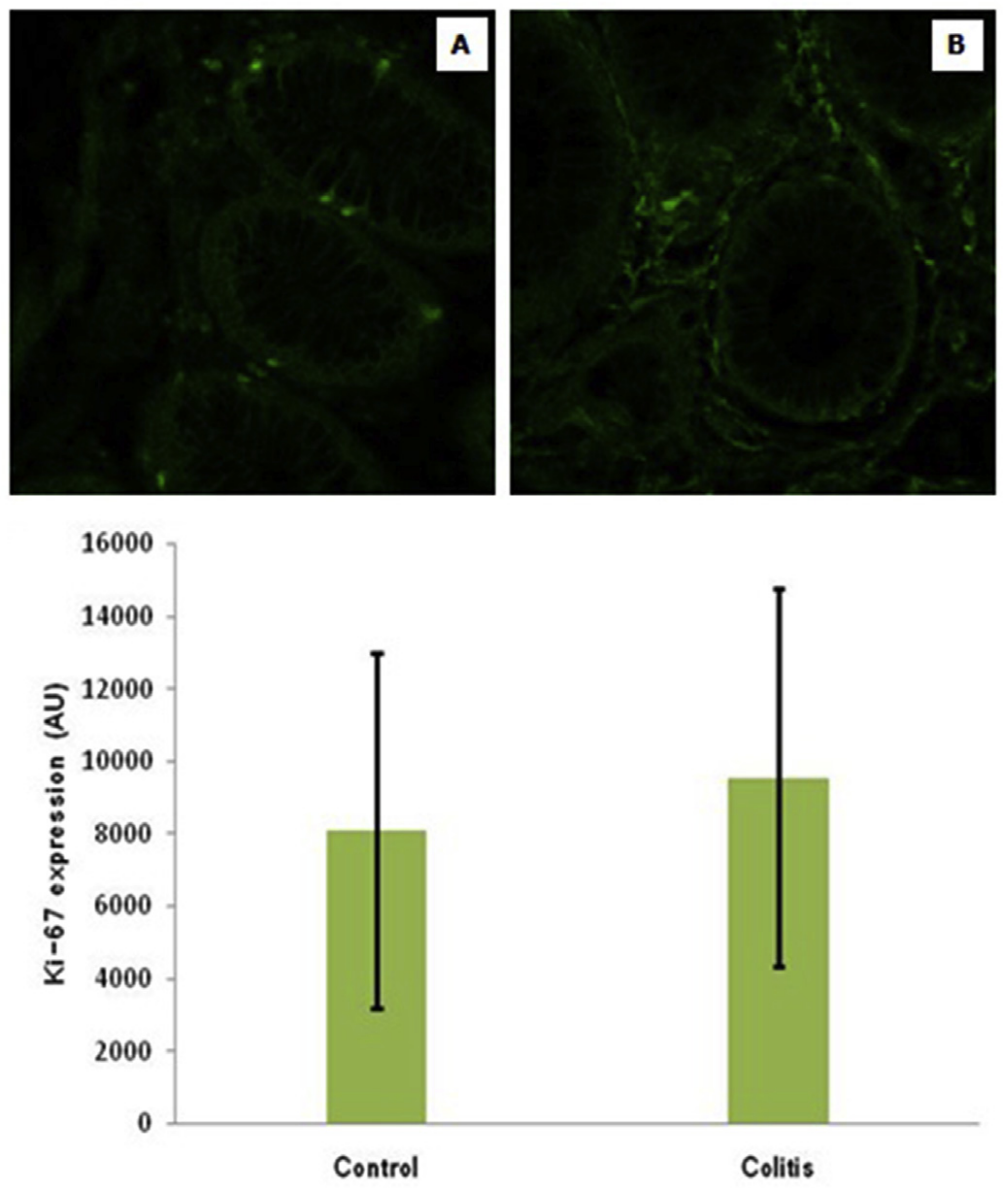
Representative micrograph of Ki-67 expression in healthy colon tissue (A) and colitis patients (B). (FITC staining at 400x magnification by a confocal laser scanning microscope) (Top figure). The expression of ki-67 in all groups. Note: Value are presented as mean ± standard of deviation; AU: arbitrary units (Bottom figure).

Fig. 1 presents the caspase-3 expressions in all groups. Caspase-3 expression decreased significantly in colitis patients compared to the control group (*p* < 0.05). Meanwhile, there were no differences in Ki-67 expressed in various groups (*p* > 0.05), as shown in Fig. 2.

**Fig. 3:**
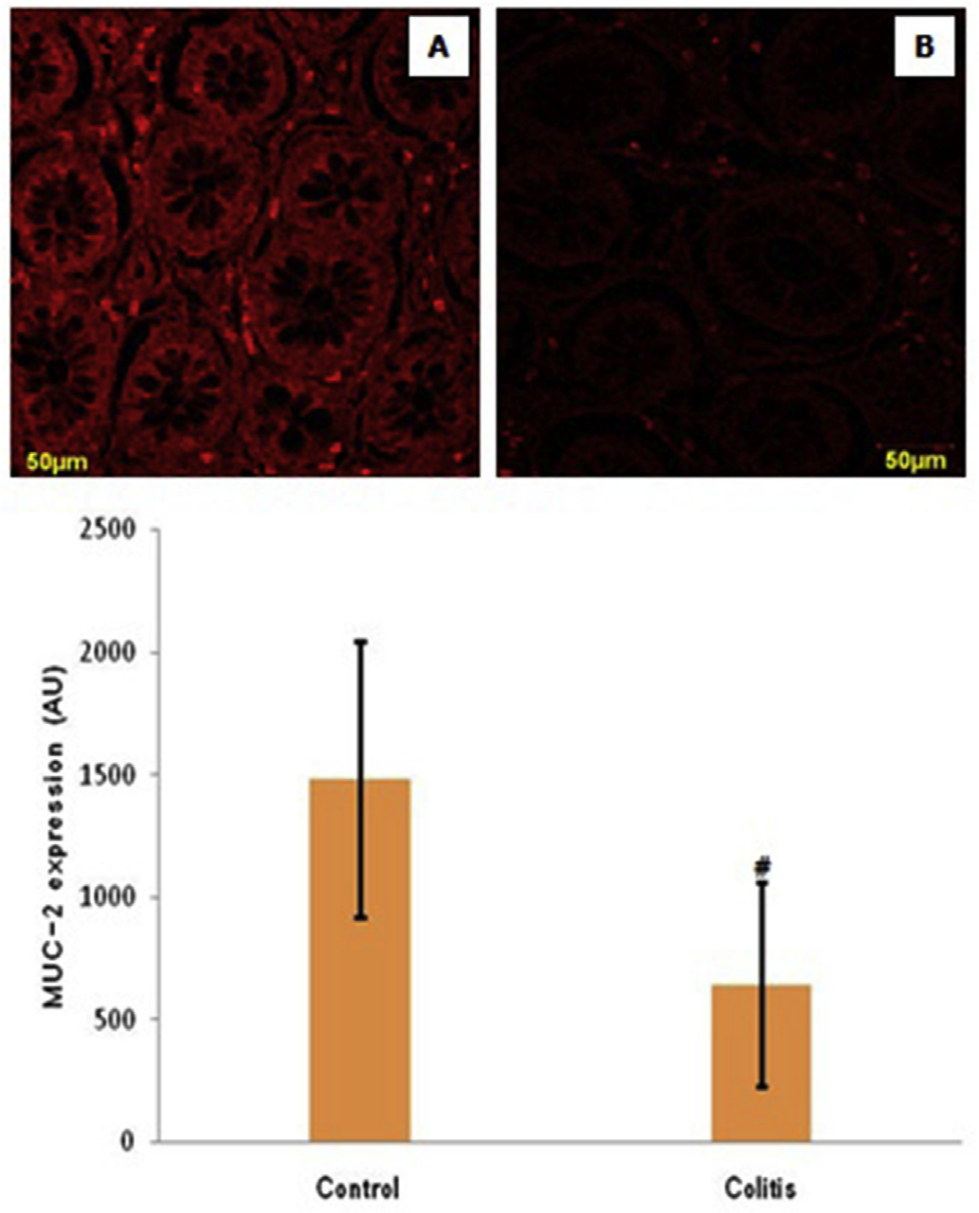
Representative micrograph of MUC-2 expression in healthy colon tissue (as a control) (A) and colitis patients (B). (Rhodamin staining at 400x magnification by a confocal laser scanning microscope) (Top figure). The expression of MUC-2 in both groups. Note: Value are presented as mean ± standard of deviation; AU: arbitrary units; ^#^: *p* < 0.05 in comparison with the control group (Bottom figure).

Expressions of MUC-2 in various groups can be seen in Fig. 3. MUC-2 expression decreased significantly in the colitis group compared to the control group (*p* < 0.05).

Table 1 shows the MUC2 mutations and NF-κB expression between various groups. The expression of MUC2 mutations and the NF-κB expression in the colitis group did not differ significantly compared to the control group (*p* > 0.05).

**Table 1:** The MUC-2 mutation and NF-κB expression in all groups.

	Control (n = 11)	Colitis (n = 6)	*p* value
FISH MUC-2 (AU)	10412.809 ± 4773.534	13110.551 ± 8702.766	*p* > 0.05
NF-κB (AU)	19588.144 ± 11975.148	16419.078 ± 9752.170	*p* > 0.05

Note: Value presented as mean ± standard of deviation; FISH: fluorescence in situ hybridization; AU: arbitrary units.

**Fig. 4:**
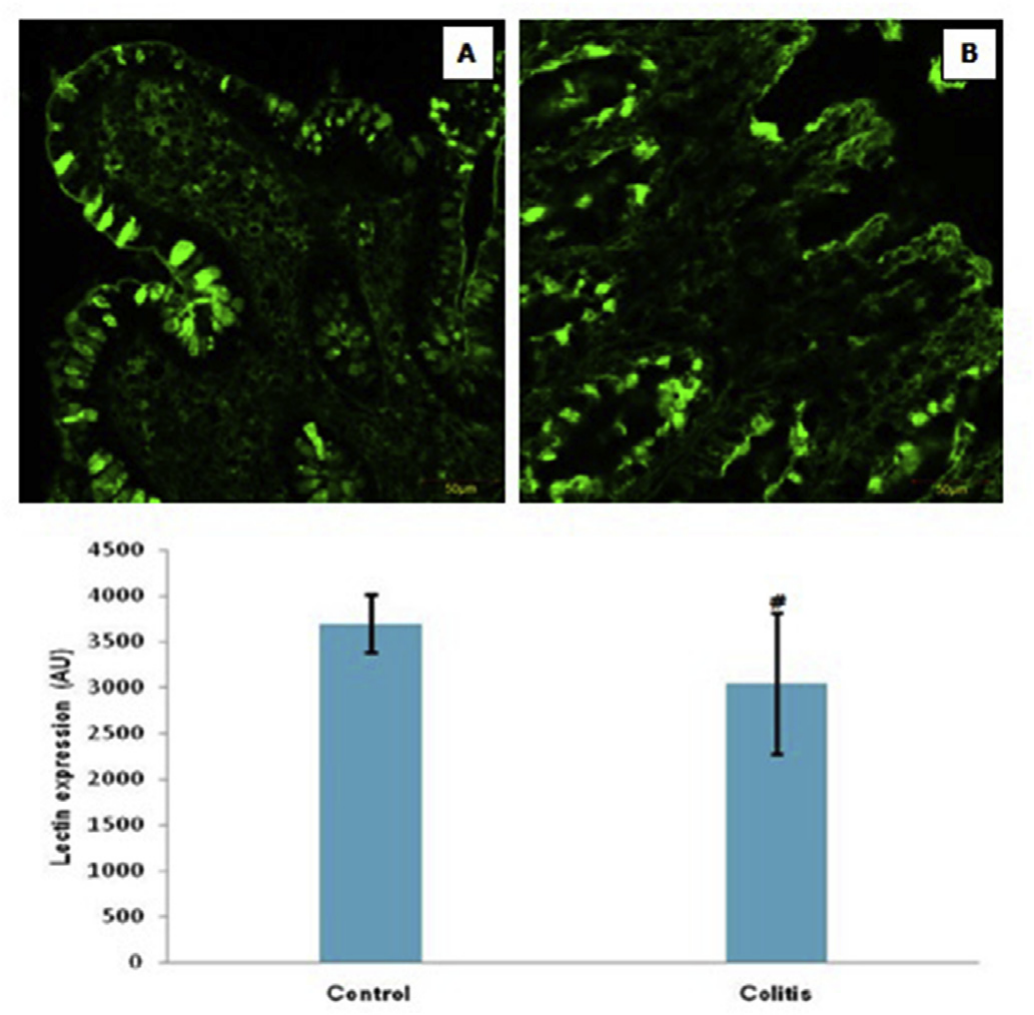
Representative micrograph of lectin expression in healthy colon tissue (as a control) (A) and colitis patients (B). (FITC staining at 400x magnification by a confocal laser scanning microscope) (Top figure). The expression of lectin in both groups. Note: Value are presented as mean ± standard of deviation; AU: arbitrary units; ^#^: *p* < 0.05 in comparison with the control group (Bottom figure).

The expression of the lectin can be seen in Fig. 4. The expression of the lectin in colitis was significantly lower than the control group (*p* > 0.05).

## 4 Discussion

In this study, caspase-3 expression was significantly lower in the colitis group than in the control group. These findings indicate that there is an apoptotic defect in colitis compared to controls. This study contrasts with previous findings that found an increase in apoptosis assessed by TUNEL in colitis patients [23]. In rat colitis models due to DSS induction also found an increase in caspase-3 expression [24]. The results of this study extend the previous findings that apoptosis resistance colitis [25]. The decrease in caspase-3 expression is thought to be caused by high autophagy activity which limits apoptosis due to cytokine induction so that the inflammation is also minimal [26]. This is supported by the finding that NF-κB expression was not significantly different between colitis and controls. Apoptotic defects in colitis were not accompanied by changes in cell proliferation, as evidenced by the meaningless expression of KI-67 between colitis and control patients.

In this study, MUC-2 expression decreased significantly in colitis compared to controls. This indicates a down-regulation of MUC-2 expression in colitis compared to controls. This finding is consistent with previous studies that there was a decrease in the expression of MUC-2 in colitis compared to controls [8]. We hypothesized that this decrease in expression was caused by a decrease in production and/or an increase in degradation of MUC-2. The results of the FISH analysis showed no significant differences between colitis and controls. This leads the researcher that the decrease in MUC-2 expression is not caused by processes at the level of gene transcription, but in the process of post-translation modification. One post-translational modification is a glycan change, protecting the core protein from protease activity [14,15]. On the other hand, there was no significant difference in NF-κB activity in colitis patients compared to controls. Thus, in this study the decrease in expression of MUC-2 in colitis patients did not involve an increase in inflammation and changes in the level of gene transcription. The degree of inflammation in colitis is related to glycan changes [16]. This study also found a significant decrease in lectin expression in colitis compared to controls. This finding indicates that in colitis there is a decrease in the location of interactions between carbohydrates (glycan) and lectins. This confirms that the decrease in MUC2 is caused by the post-translation process, one of which is due to a decrease in the expression of the lectin. Decreasing the expression of the lectin will reduce the interaction with glycan, so that the glycan protective effect on protease activity decreases. Furthermore, MUC2 will be degraded by proteases so that it decreases.

The limitations of this study include the small number of samples and no identification of the causes of colitis. This will be a concern in future studies.

It was concluded that in colitis there was a change in MUC2 expression due to changes in lectins accompanied by apoptotic defects.

## Conflicts of interest

All the authors state that there is no conflict of interest in the research or publication of this article.

## Appendix A Supplementary data

Application Application

## References

[1] Choi E.Y.K., Appelman H.D. (2017). Chronic colitis in biopsy samples. Is it inflammatory bowel disease or something else?. Surg. Pathol.

[2] Maloy K.J., Powrie F. (2011). Intestinal homeostasis and its breakdown in inflammatory bowel disease. Nature.

[3] Neurath M.F. (2014). Cytokines in inflammatory bowel disease. Nat. Rev. Immunol..

[4] Lonnfors S., Vermeire S., Greco M., Hommes D., Bell C., Avedano L. (2014). IBD and health-related quality of life – discovering the true impact. J Crohns Colitis.

[5] Burisch J., Jess T., Martinato M., Lakatos P.L. (2014). The burden of inflammatory bowel disease in Europe. J Crohns Colitis.

[7] Ng S.C., Shi H.Y., Hamidi N., Underwood F.E., Tang W., Benchimol E.L., Panaccione R., Ghosh S., Wu J.C.Y., Chan F.K.L., Sung J.J.Y., Kaplan G. (2018). Worldwide incidence and prevalence of inflammatory bowel disease in the 21st century: a systematic review of population-based studies. Lancet.

[8] Niv Y. (2016). Mucin gene expression in the intestine of ulcerative colitis patients: a systematic review and meta-analysis. Eur. J. Gastroenterol. Hepatol..

[9] Hinoda Y., Akashi H., Suwa T., Itoh F., Adachi M., Endo T., Satoh M., Xing P.X., Imai K. (1998). Immunohistochemical detection of MUC2 mucin core protein in ulcerative colitis. J. Clin. Lab. Anal..

[10] Doroveyef A.E., Vasilenko I.V., Rassokhina O.A., Kondratiuk R.B. (2013). Mucosal barrier in ulcerative colitis and Crohn's disease. Gastroenterol. Res. Pract..

[11] Tytgat K.M.A.J., van der Wal J.W.G., Einerhand A.W.C., Buller H.A., Dekke J. (1996). Quantitative analysis of MUC2 synthesis in ulcerative colitis. Biochem. Biophys. Res. Commun..

[12] Moulari B., Beduneau A., Pellequer Y., Lamprecht A. (2014). Lectin-decorated nanoparticles enhance binding to the inflamed tissue in experimental colitis. J. Control. Release.

[13] de Juan LV RecioVG., Lopez P.J., Juan T.G., Cordoba-Diaz M., Cordoba-Diaz D. (2017). Pharmaceutical applications of lectins. J. Drug Deliv. Sci. Technol..

[14] Johansson M.E.V., Hansson G.C. (2013). Mucus and the goblet cell. Dig. Dis..

[15] Bergstorm K., Liu X., Zhao Y., Gao N., Wu Q., Song K., Cui Y., Li Y., McDaniel J.M., McGee S., Chen W., Huycke M.M., Houchen C.W., Zenewics L.A., West C.M., Chen H., Braun J., Fu J., Xia L. (2016). Defective intestinal mucin-type o-glycosylation causes spontaneous colitis-associated cancer in mice. Gastroenterology.

[16] Larsson J.M., Karlsson H., Crespo J.G., Johansson M.E., Eklund L., Sjövall H., Hansson G.C. (2011). Altered O-glycosylation profile of MUC2 mucin occurs in active ulcerative colitis and is associated with increased inflammation. Inflamm. Bowel Dis..

[17] Dierckx T., Verstockt B., Vermiere S., Van Weyenbergh J. (2018). GlycA, a nuclear magnetic resonance spectroscopy measure for protein glycosylation, is a viable biomarker for disease activity in IBD. J Chrons Colitis.

[18] Cohen B.L., Sachar D.B. (2017). Update on anti-tumor necrosis factor agents and other new drugs for inflammatory bowel disease. BMJ.

[19] Hendy P., Hart A., Irving P. (2016). Anti-TNF drug and antidrug antibody level monitoring in IBD: a practical guide. Frontline Gastroenterol..

[20] Arumugam S., Thandavarayan R.A., Pitchaimani V., Karuppagounder V., Harima M., Nishizawa Y. (2014). Prevention of DSS induced acute colitis by Petit Vert, a newly developed function improved vegetable, in mice. Pharma Nutr..

[21] Neubauer K., Wozniak-Stolarska B., Krzystek-Korpacka M. (2018). Peripheral lymphocytes of patients with inflammatory bowel disease have altered concentrations of key apoptosis players: preliminary results. BioMed Res. Int..

[22] Kania N., Setiawan B., Widjadjanto E., Nurdiana N., Widodo M.A., Kusuma H.M.S.C. (2014). Subchronic inhalation of coal dust particulate matter 10 induces bronchoalveolar hyperplasia and decreases MUC5AC expression inmale Wistar rats. Exp. Toxicol. Pathol..

[23] Souza H.S.P., Tortori C.J.A., Castelo-Branco M.T.L. (2005). Apoptosis in the intestinal mucosa of patients with inflammatory bowel disease: evidence of altered expression of FasL and perforin cytotoxic pathway. Int. J. Colorectal Dis..

[24] Sreedhar R., Arumugam S., Karuppagounder V., Thandavarayan R.A., Giridharan V.V., Pitchaimani V., Afrin M.R., Harima M., Nakamura T., Nakamura M., Suzuki K., Watanabe K. (2015). Jumihaidokuto effectively inhibits colon inflammation and apoptosis in mice with acute colitis. Int. Immunopharmacol..

[25] Pott J., Kabat A.M., Maloy K.J. (2018). Intestinal epithelial cell autophagy is required to protect against tnf-induced apoptosis during chronic colitis in mice. Cell Host Microbe.

[26] Fayad R., Brand M.I., Stone D., Keshavarzian A., Qiao L. (2006). Apoptosis resistance in ulcerative colitis: high expression of decoy receptors by lamina propria T cells. Eur. J. Immunol..

